# Antitumor activity of a 5T4 targeting antibody drug conjugate with a novel payload derived from MMAF via C‐Lock linker

**DOI:** 10.1002/cam4.2066

**Published:** 2019-03-07

**Authors:** Baoying Shi, Min Wu, Zhaohui Li, Zhangming Xie, Xiaoyue Wei, Jiansheng Fan, Yingchun Xu, Ding Ding, Sajid Hamid Akash, Shuqing Chen, Sheldon Cao

**Affiliations:** ^1^ College of Pharmaceutical Sciences, Institute of Drug Metabolism and Pharmaceutical Analysis and Zhejiang Provincial Key Laboratory of Anti‐Cancer Drug Research Zhejiang University Hangzhou China; ^2^ Zova Biotherapeutics Inc Fuyang, Hangzhou China; ^3^ Noeantigen Therapeutics (HangZhou) Co., Ltd Hangzhou China

**Keywords:** antibody drug conjugate, antitumor activity, C‐Lock linker, MMAF analogue, ZV0508

## Abstract

Antibody‐drug conjugates (ADCs) belong to a promising class of biopharmaceuticals in which target‐killing of tumor cells was achieved by marrying the potency of the cytotoxic payload with the tumor specificity of the antibody. Here we developed a novel ADC (ZV0508) that targets 5T4 oncofetal antigen, which is overexpressed in many carcinomas on both bulk tumor cells and cancer stem cells. A novel cytotoxic payload called Duostatin‐5 (Duo‐5) which was derived from monomethyl auristatin F (MMAF) was attached to a 5T4 targeting antibody (ZV05) by interchain cysteine cross‐linking conjugation via a disubstituted C‐Lock linker. We have investigated the antitumor efficacy of ZV0508 by in vitro and in vivo studies, and compared its antitumor activity with ZV05‐mcMMAF (ZV0501), in which MMAF was linked via a conventional noncleavable maleimidocaproyl linker. As results, ZV0508 exhibited ideal antiproliferative effects through blocking cell cycle and inducing cell apoptosis. The in vivo studies revealed that both ZV0501 and ZV0508 exhibited excellent antitumor activities even at a single dose. Although ZV0508 was inferior to ZV0501 in vitro, it elicited more durable antitumor responses than ZV0501 in vivo. The superior in vivo activity of ZV0508 may be due to the combined use of the disubstituted C‐Lock linker and the novel payload Duo‐5, resulting in a more stable and potent ADC. Taken together, these data suggest ZV0508 is a worthy candidate for the treatment of 5T4 positive cancers.

## 1 INTRODUCTION

The concept of antibody‐drug conjugate (ADC) is not recent, the cytotoxic agent is specifically delivered to the tumor sites by conjugating to the tumor‐associated antigen‐targeting monoclonal antibody via a chemical or biological linker.^1^ Till now, four ADCs have been approved by The Food and Drug Administration namely Adcetris,^2^ Kadcyla,^3^ Mylotarg,^4^ and Besponsa.^5^ The expectation of an ADC is that it maintains stability and nontoxicity in the blood circulation, while releasing the payload in an active form upon selective recognization of the antigens expressed on the tumor cell surface and internalization of the complex into the cancer cell. Thus sufficient quantity of payloads will accumulate in the tumor to exert a powerful tumor killing effect, meanwhile dose‐limiting off‐target toxicities are under reasonable control.^6^, ^7^ Summaries of ADCs approved or in late clinical development revealed that a vast majority of payloads used are anti‐mitotic or DNA damaging agents. Monomethyl auristatin E (MMAE), monomethyl auristatin F (MMAF), maytansinoid DM1 (DM1), and maytansinoid DM4 (DM4) are the most representative anti‐mitotic agents in use.^8^, ^9^ MMAF has attenuated membrane translocation capacity, less potent, higher maximal tolerated dose, and much higher aqueous solubility as compared with MMAE.^10^ At least six ADCs utilizing MMAF as payload had progressed to clinical trials by 2014.^9^

5T4 oncofetal glycoprotein, also known as trophoblast glycoprotein, is a 72 kDa N‐glycosylated transmembrane protein that is encoded on chromosome 6q14‐15.^11^, ^12^, ^13^ It is highly expressed on fetal trophoblast but lowly expressed in the normal adult tissues. In contrast, the presence of 5T4 on the cell membrane of a variety of solid carcinomas has been demonstrated.^11^, ^14^, ^15^, ^16^ Furthermore, expression of 5T4 is confirmed to be associated with advanced disease and worse clinical outcome in at least NSCLC, and gastric, ovarian, and colorectal carcinomas. Thus 5T4 is suggested to be an ideal target for ADC therapeutics. Several ADCs targeting 5T4 have been investigated so far.^21^, ^22^, ^23^, ^24^ A1mcMMAF, which was obtained by linking a humanized anti‐5T4 antibody to MMAF via a conventional noncleavable maleimidocaproyl (mc) linker, has proven excellent in vivo antitumor activity alone or by combination with PI3K/mTOR inhibitors or taxanes preclinically.^22^, ^25^ In a dose escalating phase I trial, the maximum tolerated dose of A1mcMMAF was determined to be 4.34 mg/kg with a major dose‐limiting toxicity of ocular toxicity. However, no objective responses were observed probably because patients were unselected for 5T4 expression.^26^

Researchers have reached a consensus that ADCs with better homogeneity are more favorable, based on the recent advances in ADC technology which have demonstrated that drug‐antibody ratio (DAR) and its distribution critically impact PK/PD, efficacy and toxicity of ADCs.^27^ Conventional cysteine conjugation minimizes ADC heterogeneity relative to lysine conjugation because only up to eight reactive cysteine thiol groups are released by partial reduction of four antibody inter‐chain disulfide bonds. However, the mc linkers typically used for cysteine conjugation resulted in thiosuccinimide linkerages between the payload and the antibody, which are prone to undergo thiol‐exchange reactions resulting in premature release of the payloads from the ADCs. Efforts have been made to address the instability issue of maleimide‐based ADCs by various linker modification favoring self‐hydrolysis of the thiosuccinimide ring.^28^, ^29^ Cysteine rebridging, a recently developed alternative to conventional thiol‐maleimide conjugation, uses disubstituted cores such as dibromomaleimide^30^, ^31^ and dibromopyridazinedione^32^ to cross‐link two interchain cysteines, thereby affording a rebridged antibody. This conjugation method provides many advantages in terms of structural stability, heterogeneity, and better‐controlled DAR, which in turn results in improved pharmacokinetics, superior efficacy, and reduced toxicity.^30^, ^33^, ^34^ In addition, no antibody engineering or conjugation site optimization is required, as compared with other site‐specific conjugation methods which mainly focus on antibody modification, like introduction of cysteine mutations or nonnatural amino acids.

**Figure 1:**
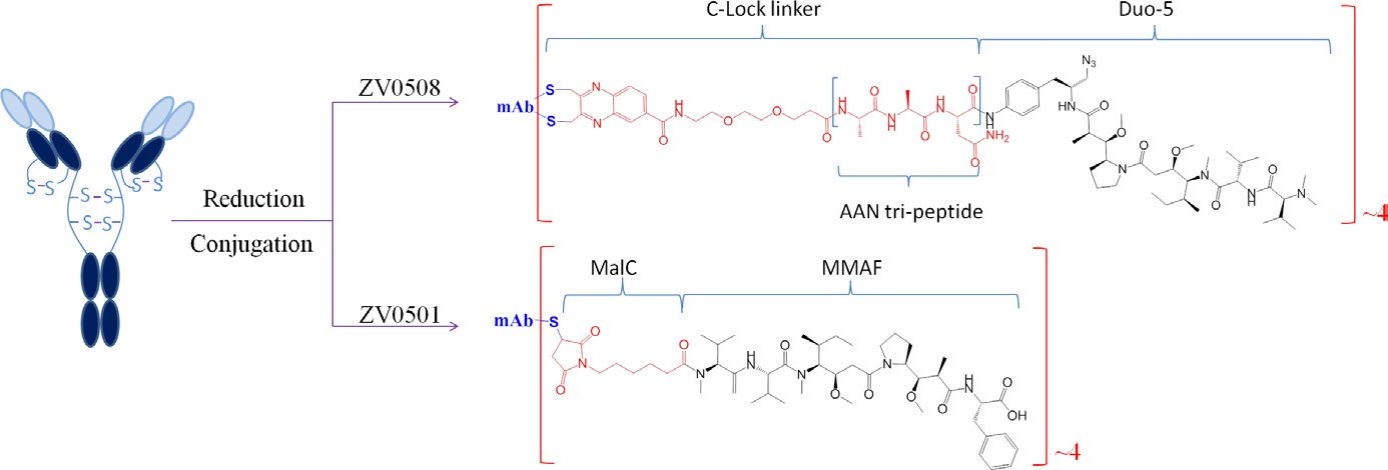
Structure illustration of ZV0508 and ZV0501

In order to develop an efficient 5T4‐targeting ADC, we conjugate a MMAF derivative named Duostatin‐5 (Duo‐5) to a 5T4‐targeting monoclonal antibody (ZV05) using a proprietary C‐Lock™ conjugation method (Figure 1), which is a similar cysteine rebridging method in essence. The antitumor efficacy of ZV0508 was investigated by detailed in vitro and in vivo studies, and its antitumor activity was further compared with ZV0501 (ZV05‐mcMMAF), another ADC with MMAF linked to ZV05 via a conventional noncleavable mc linker (Figure 1). The results presented suggested ZV0508 is a worthy candidate for the treatment of 5T4 positive cancers.

## 2 MATERIALS AND METHODS

### 2.1 Drugs and cell lines

ZV05 was a human monoclonal antibody, whose Fab was acquired by phase display. ZV0501 was produced by conjugating mc‐MMAF to ZV05 using the method described previously.^22^ ZV0508 and control ADC (targeting CD33) were produced as follows. Antibody (ZV05, or control antibody) was buffer exchanged into 50 mmol/L phosphate‐buffered solution (PBS) + 4 mmol/L EDTA, pH 7.0, and diluted to a final concentration of 5‐10 mg/mL. 10× molar equivalents of TCEP were added to the antibody. The reaction mixture was incubated at 37°C until 8 mole free thiols per mole antibody (free thiols/Ab) was detected. TCEP was then removed by ultrafiltration using 50 mmol/L PBS, 4 mmol/L EDTA, pH 7.0. 4.5× to 5× molar equivalents of drug‐linker were added from a freshly prepared 10 mmol/L stock solution in 60% acetonitrile/water. The reaction was mixed on a rotator gently at room temperature and terminated once free thiols/Ab was below 0.5. The crude ADC was buffer exchanged into PBS to remove unconjugated payloads. ZV0508 was primarily characterized by HIC‐HPLC and CE‐SDS analysis (Figures S1 and S2).

Colorectal carcinoma Lovo, pancreatic carcinoma BxPC‐3, prostatic carcinoma DU 145, and breast carcinoma MDA‐MB‐468 were obtained from Cell Bank of the Chinese Academy of Sciences (Shanghai, China), and hepatoma carcinoma HepG2 and lymphoma Romas were purchased from American Type Culture Collection (ATCC, San Francisco, CA, USA).

### 2.2 Affinity of ZV05 and ZV0508 to 5T4 extracellular domain

Ninety‐six‐well plates were coated with human 5T4 protein (1 μg/mL) and incubated at 4°C overnight. Plates were blocked with PBS + 5% skim milk at 37°C for 2 hours. Next, ZV05 or ZV0508 with concentrations ranging from 0.051 to 3000 ng/mL was added to the wells and incubated at 37°C for 2 hours. The wells were washed, and HRP‐labeled goat anti‐human IgG (H+L) polyclonal antibody was added and incubated for 2 hours at 37°C. After washing, TMB was added to each well, and the reaction was quenched after a 20 minutes‐incubation at 37°C. The optical density was measured at 450 nm.

### 2.3 Binding of ZV05 and ZV0508 to cancer cell lines

The binding affinity of ZV05 and ZV0508 to 5T4‐positive human cancer cell lines was determined by flow cytometry. 3 × 10^5^ cells were incubated with ZV05 or ZV0508 in PBS + 1% bovine serum albumin (w/v) for 30 minutes on ice. After incubation, cells were washed twice with PBS and then incubated with fluorescein isothiocyanate (FITC)‐labeled goat anti‐human IgG (H+L) polyclonal antibody at 1:300 for 30 minutes on ice. After washing, cells were examined on a Beckman Coulter Cytomics FC500 Flow Cytometer. Data analysis was performed using CXP analysis 2.2 (Beckman Coulter) and the geometric mean of fluorescence intensity ratio (MFI) of each cell line was determined.

### 2.4 Internalization and microscopy of ZV05 and ZV0508

To determine the internalization of cell surface‐bound ZV05 or ZV0508, cells were saturated with excess ZV05 or ZV0508 (10 μg/mL) in culture medium at 4°C for 30 minutes. After washing, cells were incubated at either 4°C or 37°C for required hours to drive internalization. The internalization reaction was stopped by washing with cold PBS, and then cells were incubated with FITC‐labeled goat anti‐human IgG (H+L) polyclonal antibody at 1:300 for 30 minutes on ice. After washing, cells were analyzed by flow cytometry. The internalization percentage of antibody or ADC at each time point was determined by MFI using the following formula: % internalized = (total surface bound (4°C) − total surface bound (37°C))/total surface bound (4°C) × 100%.

The dynamic internalization process of ADC and cell surface 5T4 antigen in MDA‐MB‐468 cells was determined. Cells were saturated with 10 μg/mL of ZV0508 at 4°C for 30 minutes, washed to remove unbound ADC, and then divided into two groups (specified one sample as 0 hours, MFI represented the initial amount of surface antigen and bound ADC as well). For the first group, which represented the dynamic internalization process of ADC, cells were incubated for 1, 2, 4, 6, 8, 12, and 24 hours at 37°C, then incubated with goat anti‐human IgG H&L (DyLight® 488) polyclonal antibody at 1:200 for 30 minutes on ice. After washing, cells were examined on a Beckman Coulter Cytomics FC500 Flow Cytometer subsequently. For the second group, which represented the dynamic internalization process of cell surface 5T4 antigen, after being incubated for 1, 3, 7, 12, and 24 hours at 37°C, cells were subsequently incubated with ZV0508 (10 μg/mL) again in PBS + 1% bovine serum albumin (w/v) for 30 minutes on ice. After incubation, cells were washed twice with PBS, and incubated with goat anti‐human IgG H&L (DyLight® 488) polyclonal antibody at 1:200 for 30 minutes on ice. After washing, cells were examined on a Beckman Coulter Cytomics FC500 Flow Cytometer. The internalization percentage was determined by MFI as above.

For visualized internalization assay by microscopy, cells seeded at a density of 5 × 10^4^ cells/mL were treated with 10 μg/mL of ZV0508 on ice, washed and then incubated at 37°C for 0 or 6 hours. ZV0508 was detected with FITC‐labeled goat anti‐human IgG (H+L) polyclonal antibody, lysosomes with rabbit monoclonal antibody against lysosome‐associated membrane protein‐1 (LAMP‐1) followed by Cy3‐labeled goat anti‐rabbit IgG (H+L) polyclonal antibody, and nuclei with 4′,6‐diamidino‐2‐phenylindole. Cells were pictured using an Olympus IX81‐FV1000 Microscope.

### 2.5 In vitro* cytotoxicity assay*

Cells were seeded at a density of 5 × 10^3^ cells/well in 96‐well plates. After 24 hours incubation, cells were exposed to various concentrations of test articles (ZV05, ZV0508, ZV0501, and control ADC) at 37°C for 72 hours. Cell viability was determined by Cell Counting Kit‐8 (CCK‐8). The absorbance was measured at 450 nm by BioRad microplate reader (Model 680 Microp).

### 2.6 Apoptosis assay by flow cytometry and immunoblot analysis

Cells were seeded at a density of 2.5 × 10^5^ cells/mL and exposed to ZV05 or ZV0508 at various concentrations for 72 hours. The control group was incubated with medium alone. After exposure, cells were collected and stained with AnnexinV‐FITC and PI for apoptosis analysis. The percentages of apoptotic cells (AnnexinV+/PI− and/AnnexinV+/PI+) were determined by flow cytometric analysis of each population.

For immunoblot analysis, drug‐exposed cells were re‐suspended in RIPA buffer with 1 mmol/L of PMSF and shake at 4°C for 2 hours. The protein concentrations in the supernatant were determined using Nanodrop. Proteins were separated by 12% SDS‐PAGE and electro blotted onto nitrocellulose membranes (Bio‐Rad, Mississauga, ON). The membranes were blocked in PBS + 10% skim milk + 0.1% Tween‐20 at room temperature for 1.5 hours, and then incubated overnight at 4°C with rabbit anti‐PARP antibody or mouse anti‐β Actin monoclonal antibody, followed by incubation with HRP‐labeled goat anti‐rabbit IgG (H+L) polyclonal antibody or HRP‐labeled goat anti‐mouse IgG (H+L) polyclonal antibody, respectively. Protein bands were visualized with EZ‐ECL Chemiluminescence Kit for HRP.

### 2.7 Cell cycle assay

Cells were seeded at a density of 2.5 × 10^5^ cells/mL and exposed to ZV05 or ZV0508 at various concentrations for 72 hours. The control group was incubated with medium alone. After exposure, cells were collected and fixed with 70% ethanol at 4°C for 16 hours. Cells were washed and stained with PI for 30 minutes, and examined on a Beckman Coulter Cytomics FC500 Flow Cytometer.

### 2.8 Distribution of ZV05 in human tumor xenograft mouse model

ZV05 was labeled with Cyanine 5 NHS ester (Lumiprobe) according to the product manual. 1 × 10^7^ MDA‐MB‐468 cells or Romas cells were injected subcutaneously into the 6–8‐week‐old female Balb/c nude mice. When the tumors reached an average volume of 500 mm^3^, mice were injected with 5 mg/kg Cyanine 5 labeled ZV05 by tail vein injection. The control group was injected with PBS. The fluorescence distribution images were acquired by Maestro in vivo imaging system.

### 2.9 In vivo* antitumor activities of ZV0508 and ZV0501*

All the procedures related to animal handling, care, and the treatment were performed in accordance with the guidance of Association for Assessment and Accreditation of Laboratory Animal Care. For the MDA‐MB‐468 model, 6–7‐week‐old Balb/c nude female mice were inoculated subcutaneously with 5 × 10^6^ MDA‐MB‐468 tumor cells. When the average tumor volume reached 300 mm^3^, mice were divided into three groups and injected intravenously with PBS, ZV0508 (3 mg/kg), and ZV0501 (3 mg/kg) for a single dose. For the BxPC‐3 model, 4–6‐week‐old Balb/c nude male mice were inoculated subcutaneously with BxPC‐3 tumor tissue (1 mm × 1 mm × 1 mm). When the average tumor volume reached 130 mm^3^, mice were divided into three groups and injected intravenously with PBS, ZV0508 (5 mg/kg), and ZV0501 (5 mg/kg) for a single dose. For the DU 145 model, 6–8‐week‐old Balb/c nude male mice were inoculated with 5 × 10^6^ DU 145 tumor cells. When the average tumor volume reached 216 mm^3^, mice were divided into three groups and injected intravenously with PBS, ZV0508 (2 mg/kg or 5 mg/kg) for a single dose. For the Lovo model, 6–8‐week‐old Balb/c nude male mice were inoculated with Lovo tumor tissue (1 mm × 1 mm × 1 mm). When the average tumor volume reached 400 mm^3^, mice were divided into three groups and injected intravenously with PBS, ZV0508 (10 mg/kg), and ZV0501 (10 mg/kg) for a single dose.

Tumor volume was measured twice a week in two dimensions using a caliper, and the volume was expressed in mm^3^ using the formula: V = (L × W^2^)/2 where L and W are the long and short diameters of the tumor, respectively. And the body weight in each group was continuously monitored till the end of the experiment. The date of drug administration was denoted as Day 0.

## 3 RESULTS

### 3.1 Affinity of ZV05 and ZV0508 to 5T4 protein and cells

**Figure 2:**
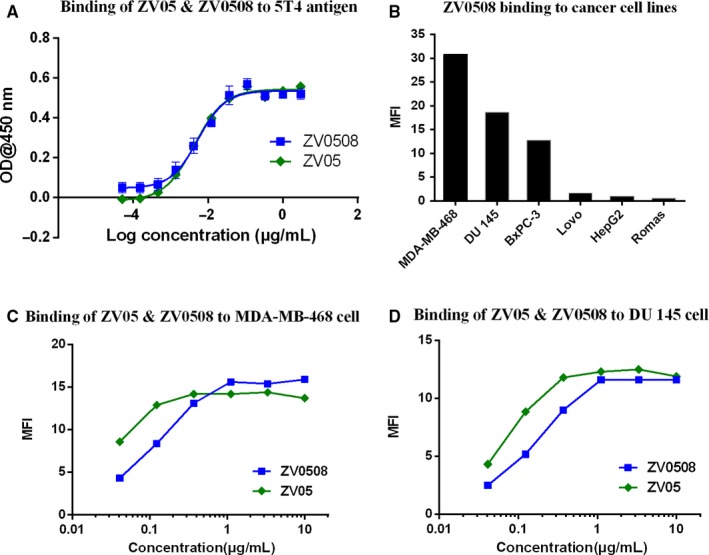
Binding affinity of ZV0508. A, Binding of ZV05 and ZV0508 to 5T4 extracelluar domain by ELISA. B, Relative 5T4 expression level on different cell lines. Cells were incubated with 10 μg/mL ZV0508 followed with FITC‐labeled goat anti‐human IgG (H+L) polyclonal antibody. C, Binding of ZV05 and ZV0508 to MDA‐MB‐468 cells by flow cytometric analysis (detected with FITC). D, Binding of ZV05 and ZV0508 to DU 145 cells by flow cytometric analysis (detected with FITC)

Binding of ZV05 or ZV0508 to 5T4 extracellular domain was determined by ELISA. As shown in Figure 2A, ZV0508 had an EC_50_ of 5.4 ng/mL, which was quite close to ZV05 (4.3 ng/mL), suggesting that the binding ability of ZV05 with 5T4 extracellular domain was not affected by conjugated Duo‐5 payload. To determine whether the binding affinity of ZV0508 to 5T4‐positive cell lines was influenced by Duo‐5 payload, we first examined the cell surface expression level of 5T4 in several cell lines (Figure 2B). As we can see, MDA‐MB‐468 cell line had the highest MFI, followed by DU 145, BxPC‐3, and Lovo cell lines in order with a moderate or low MFI, respectively. In contrast, ZV0508 bound to neither HepG2 nor Romas. The relative expression levels of 5T4 detected in the above cell lines were consistent to the data previously reported. Next, MDA‐MB‐468 and DU‐145 cell lines were chosen to assess the binding affinity of ZV0508. The result showed a slight decrease but no decisive change in the binding affinity between the naked antibody and its ADC form (Figure 2C,D).

### 3.2 Determination of internalization in 5T4‐positive cell lines

**Figure 3:**
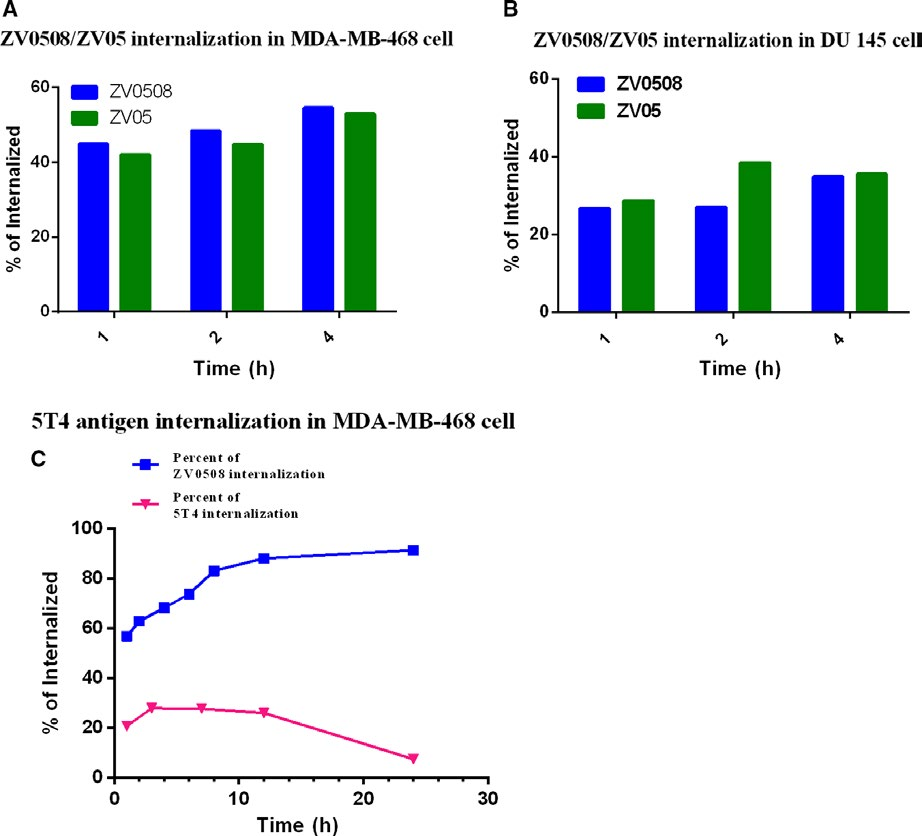
Internalization of ZV05 and ZV0508 in 5T4‐positive cells. A, Internalization of ZV05 and ZV0508 in MDA‐MB‐468 cells (detected with FITC). B, Internalization of ZV05 and ZV0508 in DU 145 cells (detected with FITC). C, Dynamic internalization process of cell surface 5T4 antigen in MDA‐MB‐468 cells (detected with DyLight® 488)

The internalization rates of ZV05 and ZV0508 were determined in two 5T4‐positive cell lines (MDA‐MB‐468 and DU 145). As shown in Figure 3A,B, the internalization of ZV05 and ZV0508 increased with time. The internalization rate of the antibody was not influenced by conjugation, since ZV05 and ZV0508 had similar internalization rates in the same cell line, although the internalization rate varied a little between DU 145 and MDA‐MB‐468 cell lines.

We also characterized the dynamic internalization process of ADC and cell surface 5T4 antigen in MDA‐MB‐468 cells (Figure 3C). As we can see, the % internalization of surface bound ZV0508 gradually increased within 12 hours, while the % internalization of surface antigen was basically maintained at around 20%. By 24 hours, the internalization of ZV0508 was almost completed with a calculation of 91% of the internalized ZV0508 (which might also include a certain amount of shedding ZV0508 from the cell surface), and the percentage of surface antigen was recovered to nearly the initial level accordingly.

**Figure 4:**
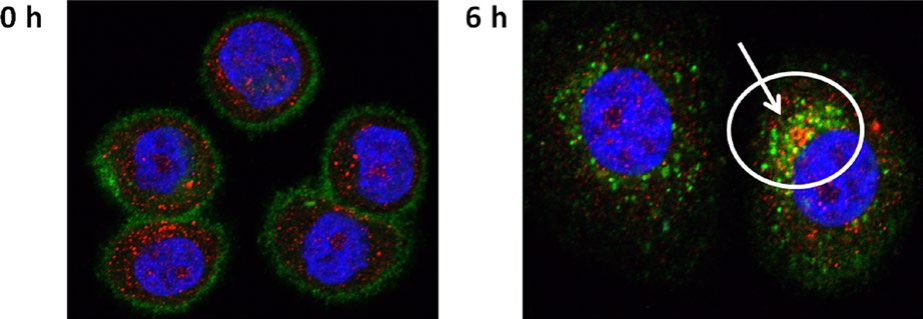
Subcellular localization of ZV0508 in MDA‐MB‐468 cells. Nuclei were stained with 4′,6‐diamidino‐2‐phenylindole (blue). ZV0508 was detected with FITC‐labeled secondary antibody (green), and lysosome‐associated membrane protein‐1 (LAMP‐1) was detected with Cy3‐labeled (red) secondary antibody. Arrow indicated co‐localization (yellow) of ZV0508 with lysosomes

The internalization of ZV0508 in MDA‐MB‐468 cells was further pictured using the confocal microscope (Figure 4). ZV0508 only bound to the membrane of MDA‐MB‐468 cells when incubated on ice, and that 6 hours after subsequent transfer to 37°C, ZV0508 was found in the lysosomes. The above results suggested that ZV0508 binds to 5T4 positive cells, internalizes and traffics to the lysosomes where the active drug payload will be released by enzymatic hydrolysis.

### 3.3 Determination of apoptosis

**Figure 5:**
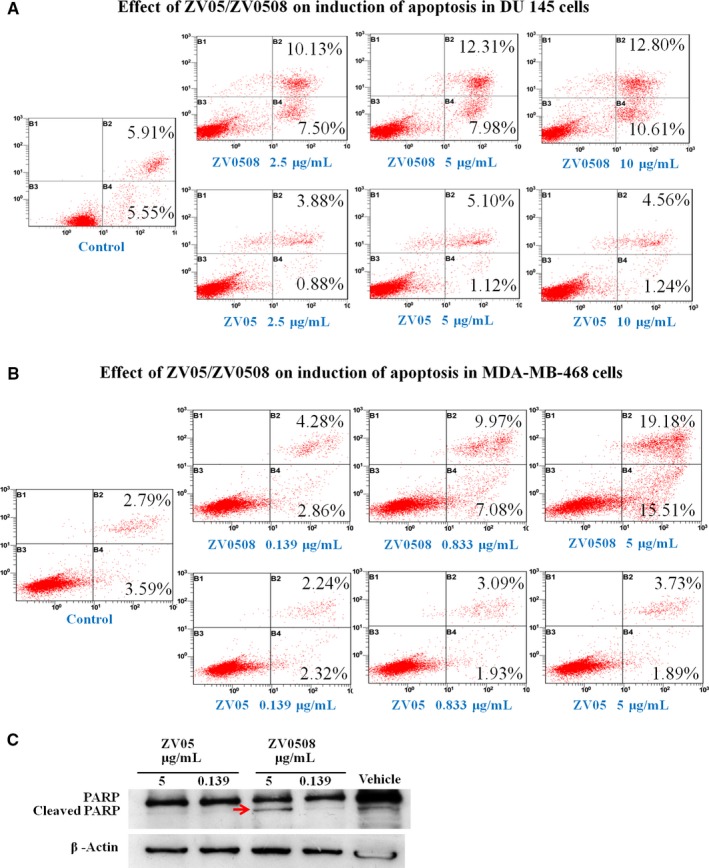
Effect of ZV05 and ZV0508 on induction of cell apoptosis. A, DU 145 and B, MDA‐MB‐468 cells were exposed to medium, ZV05 or ZV0508 at indicated concentrations. Damaged cells: Annexin V‐FITC negative/PI positive (B1). Late apoptotic cells: Annexin V‐FITC positive/PI positive (B2). Live cells: Annexin V‐FITC negative/PI negative (B3). Early apoptotic cells: Annexin V‐FITC positive/PI negative (B4). C, PARP cleavage analysis

Induction of apoptosis in DU 145 and MDA‐MB‐468 cells was determined, as shown in Figure 5A,B. The percentage of apoptosis cells in ZV0508‐treated groups increased as the concentration of ZV0508 increased, while ZV05‐treated groups concentration‐independently showed a low percentage of apoptosis cells (nearly the same as the control). In addition, apoptosis‐related proteins PARP and cleaved PARP were also determined (Figure 5C). When apoptosis happens, protein PARP is cleaved into two subunits. Cleaved PARP was clearly detected in MDA‐MB‐468 cells after exposure to 5 μg/mL of ZV0508 for 72 hours when compared with that of ZV05 and medium control.

### 3.4 Determination of cell cycle in 5T4‐positive cell lines

**Figure 6:**
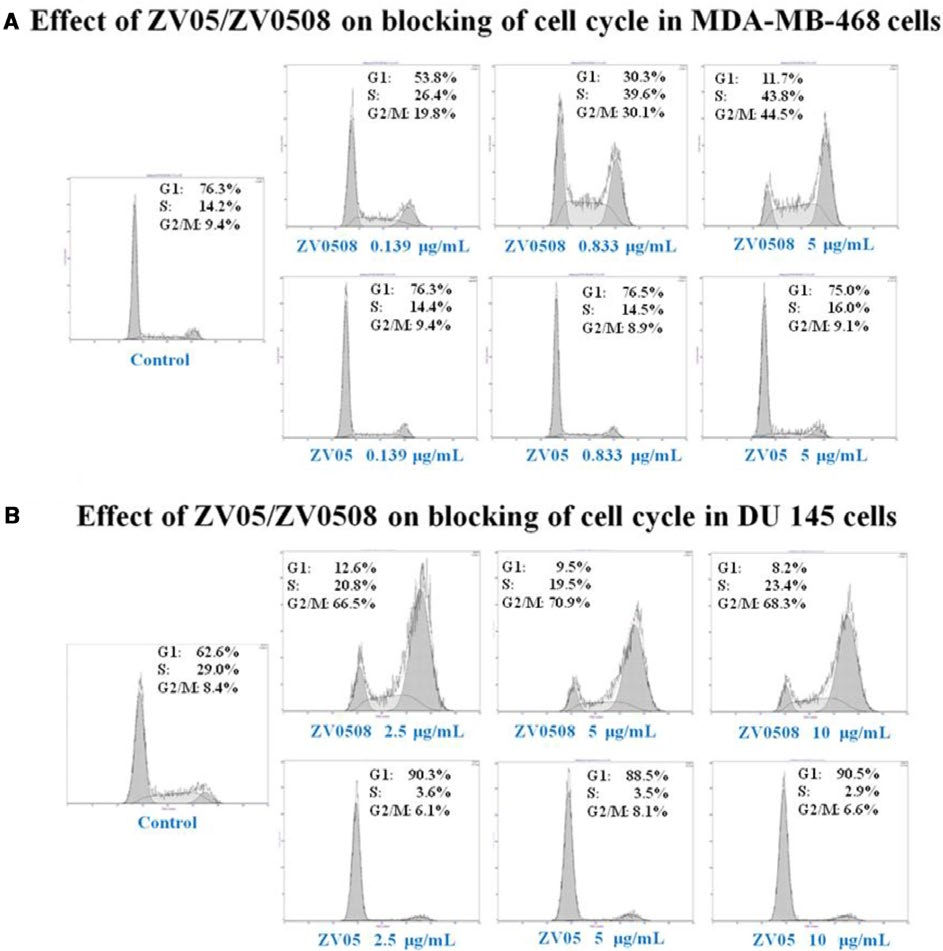
Effect of ZV05 and ZV0508 on blocking of cell cycle. A, MDA‐MB‐468 and B, DU 145 cells were exposed to medium, ZV05 or ZV0508 at indicated concentrations

Blocking of cell cycle in MDA‐MB‐468 and DU 145 cells was determined. The percentages of each cell cycle phase in different drug‐treated groups are shown in Figure 6A,B. The pattern of cell cycle in ZV05‐treated groups was concentration‐independent, and similar to the control group, representing that cell cycle was undisturbed by the naked antibody. An obvious concentration‐dependent increase of G2/M‐phase cells was observed in ZV0508‐treated groups. In MDA‐MB‐468 cell line, cell percentage of G2/M‐phase increased from 19.8% to 30.1% and 44.5% when ZV0508 concentration increased from 0.139 to 0.833 μg/mL and 5 μg/mL. Cell cycle arrested in G2/M phase was also seen in ZV0508‐treated DU 145 cell line. Overall, these results have demonstrated that ZV0508 causes cell cycle arrest at G2/M phase and induces cell apoptosis.

### 3.5 Determination of cytotoxicity

**Figure 7:**
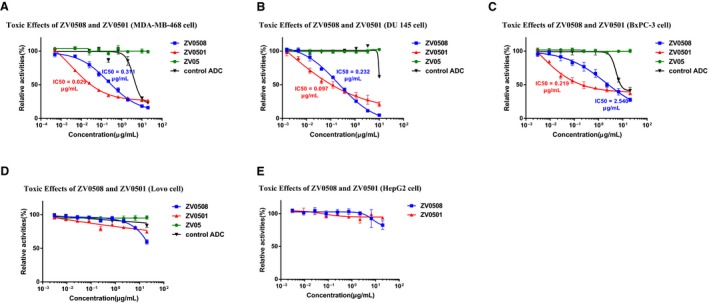
Antiproliferative efficacies of ZV0508 and ZV0501 by CCK‐8 assay on (A) MDA‐MB‐468, (B) DU 145, (C) BxPC‐3, (D) Lovo, and (E) HepG2 cell lines. Control Antibody‐drug conjugate (ADC) targeted CD33 and had the same payload and linker as ZV0508. Cell viability (%) was expressed as mean ± SD

Cell lines with different 5T4 expression were selected to evaluate the cytotoxicities of ZV0508 and ZV0501. As shown in Figure 7, ZV05 had no cytotoxicity on 5T4‐positive cells (MDA‐MB‐468, DU 145, BxPC‐3, and Lovo). The control ADC (CD33 targeted) only showed a certain degree of cytotoxicity at higher drug concentrations, while both ZV0508 and ZV0501 exhibited much stronger cell killing capabilities on 5T4‐positive cells concentration‐dependently (Figure 7A‐D). Besides, neither ZV0508 nor ZV0501 showed a cytotoxicity to 5T4 negative cell line (Figure 7E). These results suggested that the cytotoxicity was target mediated by the antibody. Moreover, both ZV0508 and ZV0501 were much more efficient in moderate or high 5T4‐expressing cell lines (Figure 7A‐C) than in low 5T4‐expressing cell line (Figure 7D). The comparison between ZV0508 and ZV0501 revealed that the IC_50_ values of ZV0508 were about 11 times, 2.4 times, and 12 times higher than those of ZV0501 in MDA‐MB‐468 (0.311 vs 0.029 μg/mL), DU 145 (0.232 vs 0.097 μg/mL), and BxPC‐3 (2.540 vs 0.219 μg/mL), respectively.

### 3.6 Determination of the distribution of ZV05 in human tumor xenograft mouse models

**Figure 8:**
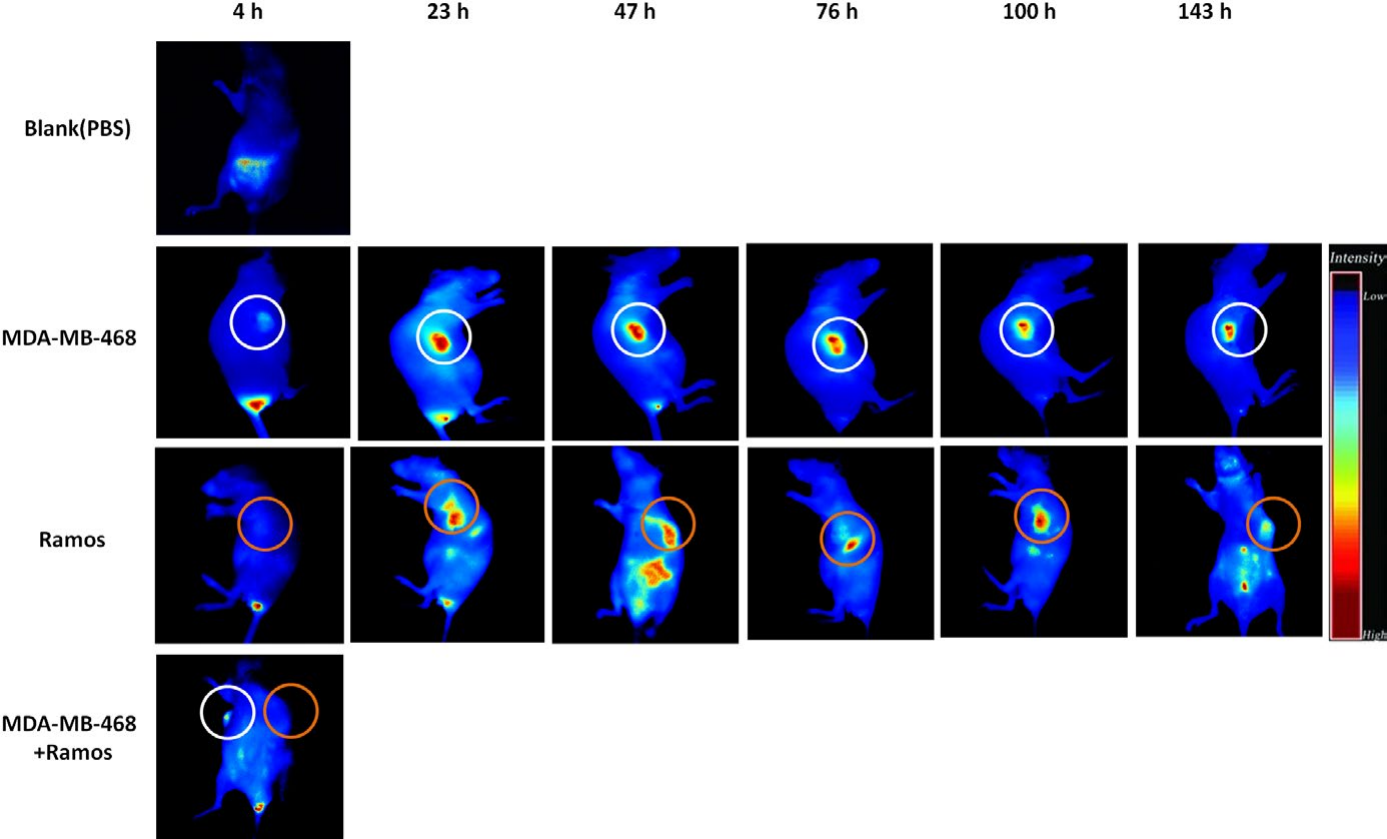
In vivo distribution of ZV05. Cyanine5‐labeled ZV05 was injected intravenously into MDA‐MB‐468 or Romas tumor‐bearing Balb/c nude mouse via tail vein (5 mg/kg). PBS was used as negative control. The circled part indicated where tumor grew

Cyanine 5 labeled ZV05 was injected into tumor‐bearing mouse for in vivo optical imaging, since the specific targeting of ZV0508 to tumor tissues is determined by the specific targeting of the naked antibody (ZV05). As we can see from Figure 8, in mouse bearing MDA‐MB‐468 cells, ZV05 accumulated rapidly in tumor site, reached a plateau and then decreased gradually. The fluorescence could still be seen at 143 hours after administration. However, the control group (mouse bearing 5T4‐negative cell line Romas) showed a completely different scene, in which ZV05 distributed not only in tumor site, but also in internal organs during the whole observation period. In the third group with mouse bearing MDA‐MB‐468 cells on one side and Romas cells on the other side, 4 hours after administration, more ZV05 accumulation was observed on the MDA‐MB‐468 side, compared to the Romas side. The above results clearly demonstrated the specificity of the naked antibody, illustrating the medication safety of ZV0508.

### 3.7 Determination of antitumor activities of ZV0508 and ZV0501 in vivo

**Figure 9:**
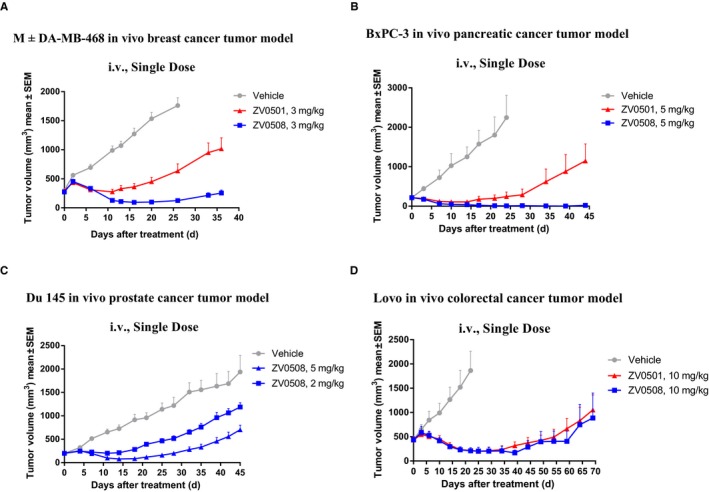
In vivo efficacy of ZV0508 and ZV0501 in human tumor xenograft mouse models. A, MDA‐MB‐468; B, BxPC‐3; C, DU 145, and D, Lovo tumors were grown subcutaneously in Balb/c nude mice. Tumor volume was presented as mean ± SEM

The antitumor activity of ZV0508 was determined in four human tumor cell xenograft models (MDA‐MB‐468, BxPC‐3, DU 145, and Lovo), and its therapeutic efficacy was compared with ZV0501 in three (MDA‐MB‐468, BxPC‐3, Lovo) of the above four xenograft models. In MDA‐MB‐468 model, ZV0508 at 3 mg/kg showed a significant tumor regression with the tumor gradually shrinking until Day 16 and slowly regrowing after that. However, weaker tumor regression and earlier tumor recurrence were seen in ZV0501 at the same dose, although its tumor growth inhibition was significant as compared with the vehicle group (Figure 9A). In BxPC‐3 model, almost complete tumor eradication was seen with 5 mg/kg ZV0508 on Day 21 and lasted till the end of the study (Day 44). ZV0501 was relatively inferior because of the weaker tumor regression and earlier tumor recurrence similar to MDA‐MB‐468 model (Figure 9B). In DU 145 model, a dose‐dependent tumor inhibition was observed. A durable tumor regression was achieved, lasting for 18 days after administration of 5 mg/kg ZV0508 (Figure 9C). In Lovo model, although ZV0508 and ZV0501 showed much less in vitro potency, they both resulted in significant tumor regression at 10 mg/kg. Besides, the effects of ZV0508 and ZV0501 were comparable, with the same degree and rate of regression (Figure 9D). In contrast to the antitumor activity observed with ZV0508 and ZV0501, much poorer activity was observed with the control ADC (Figure S3). In summary, both ZV0508 and ZV0501 exhibited excellent antitumor activities in xenograft models with different 5T4 expression levels, while ZV0508 was superior to ZV0501 in some models.

**Figure 10:**
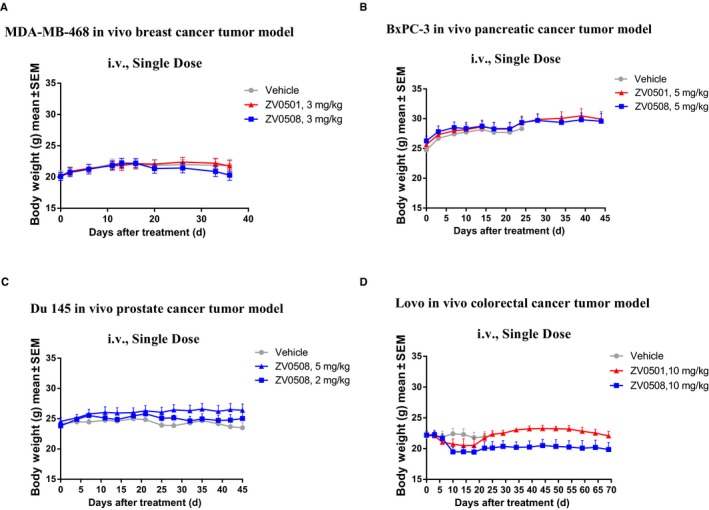
In vivo toxicity of ZV0508 in human tumor xenograft mouse models. A, MDA‐MB‐468; B, BxPC‐3; C, DU 145, and D, Lovo tumors were grown subcutaneously in Balb/c nude mice. Body weight was presented as mean ± SEM

At the same time, the weight of mice was measured to evaluate the in vivo toxicity of ZV0508 and ZV0501 (Figure 10). Slight weight loss was only observed in Lovo model when treated with 10 mg/kg of ZV0508. On the other hand, the preliminary toxicity assays revealed that no death or significant weight loss and viscera index change were observed in rats which received a single IV dose of ZV0508 at 20 mg/kg during a 3‐week observation period (Figure S4, Table S1). Besides, ZV0508 was well tolerated in cynomolgus monkeys with a single IV dose of 10 mg/kg (unpublished data). These data partially exhibited the relative safety of ZV0508.

## 4 DISCUSSION

5T4 is an attractive target for cancer therapeutics, especially with the emergence of increasing evidence showing that 5T4 is expressed on tumor‐initiating cells and associated with worse clinical outcome.^35^ Multiple therapeutic modalities targeting 5T4 have been in preclinical or clinical studies, including vaccines,^36^ CAR‐Ts,^37^ superantigens,^38^ and ADCs.^21^, ^22^, ^23^, ^24^ In this study, we developed a novel potent ADC (ZV0508) targeting 5T4 using a MMAF derivative (Duo‐5), via a proprietary interchain cysteine rebridging method named C‐Lock™ conjugation. We managed to investigate its efficacy by in vitro and in vivo studies, and compared its in vivo antitumor activity with ZV0501.

The mode of action of ZV0508 was quite typical for an ADC using an anti‐mitotic agent as payload. It was confirmed that ZV0508 bound to 5T4‐positive cells, internalized, and trafficked to the lysosomes, just like the other ADCs did.^10^, ^39^ The released active Duo‐5 would then affect cell mitosis and induce cell apoptosis. The internalization process of ZV0508 was supported by the intracellular trafficking dynamics of cell surface 5T4 antigen. The percentage of endocytic 5T4 antigen was dynamically maintained in a stable range during the internalization process of ZV0508, suggesting that the antigen continuously recycles back to the plasma membrane after cellular internalization.

The 5T4‐specific targeting of ZV0508 was confirmed from the following aspects. Firstly, ZV0508 exhibited a strong antiproliferative effect on 5T4‐positive cells but not on 5T4‐negative cells, while control ADC only showed a certain degree of cytotoxicity on 5T4‐positive cells at higher drug concentrations. And in vivo, much weaker activity was observed with the control ADC when compared with ZV0508 at the high dose of 10 mg/kg in Lovo xenograft mouse model. Secondly, in mouse bearing 5T4‐positve tumor, ZV05 accumulated rapidly in tumor site and its nonspecific accumulation in internal organs was barely seen, as compared to mouse bearing 5T4‐negative tumor, through the bioimaging analysis.

Interestingly, we found that the in vitro comparison between ZV0508 and ZV0501 was not consistent with the in vivo comparison. For the in vitro study, ZV0508 was inferior to ZV0501 when IC_50_ was adopted as the index of antitumor activity. The IC_50_ value of ZV0508 was 2‐12 times higher than that of ZV0501. However, ZV0508 outperformed ZV0501 in the in vivo antitumor studies. Specifically, stronger tumor regression and later tumor recurrence were obtained by ZV0508 in models with relative higher 5T4 expression (MDA‐MB‐468 and BxPC‐3), as compared to ZV0501 at the same dose. Of note is that no difference in the antitumor efficacy was observed between ZV0508 and ZV0501 in Lovo model which was with low 5T4‐expression. It might because of the relatively higher dose (10 mg/kg), or because they just performed equally in models with low 5T4‐expression. It remained to be explored in further studies.

The seemingly inconsistent in vitro and in vivo data is explainable. The in vitro cytotoxic assay could not mirror the real in vivo disposition of ADCs, especially the stability of ADCs in blood circulation, which is one of the main factors that may affect the efficacy. Behrens et al.^30^ reported a new ADC (trastuzumab (TRA)‐dibromomaleimide (DBM)‐MMAF) derived from interchain cysteine cross‐linking with improved tumor growth inhibition in vivo over the one derived from conventional thiol‐maleimide conjugation (TRA‐mc‐MMAF), although the superiority of TRA‐DBM‐MMAF in the in vitro assay was not observed. Further pharmacokinetic study revealed an increase in half‐life and exposure aera under curve (AUC) for TRA‐DBM‐MMAF over TRA‐mc‐MMAF. The authors believed that the improved pharmacokinetics likely contributed to the enhanced in vivo efficacy.

Theoretically, multiple factors may lead to the efficacy improvement for ZV0508 concerning its design principles: 1) the activity of the payload‐related molecule which is precisely released from the conjugate; 2) the rate of payload release and accumulation in tumor site; 3) the stability of ADC in circulation. However, the mechanism of ZV0508’s in vivo superiority over ZV0501 remained to be explored, because both linker and payload were changed. Although Duo‐5 was approximately 100 times more potent than MMAF in DU 145 and Lovo cell lines by in vitro cytotoxic assay, its in vitro efficacy dropped dramatically when a Val‐Cit linker was attached (data not provided). It might be more precise to compare their truly released payload forms. Researchers have identified cysteine‐mc‐MMAF as the major released payload form of mc‐MMAF based ADCs, but we have not yet verified the released payload form of ZV0508. So the superiority of Duo‐5 in this case is not clear so far. Based on the report that ADCs generated by cysteine rebridging method gained improved pharmacokinetics over the conventional thiol‐maleimide conjugation,^30^ and the observation in our study that the efficacy of ZV0508 was more durable than ZV0501 (more delayed and weaker tumor recurrence), we infer that ZV0508 had an improved pharmacokinetics, which might partially explain its superiority over ZV0501.

ZV0501 was the analogue of previously reported A1mcMMAF (PF‐06263507) with the same linker and payload, but it was seemingly that A1mcMMAF exerted more potent antitumor activity in MDA‐MB‐468 tumor model than ZV0501. This conflicting data may be due to differences in dosing schedule (single administration of ZV0501 in our study and Q4d×4 of A1mcMMAF in the literature). The repeated administration may allow A1mcMMAF remain its blood concentration within the therapeutic window for a longer time.

In conclusion, we tested a novel 5T4‐targeting ADC (ZV0508), which was generated by interchain cysteine rebridging conjugation method. The promising antitumor activity of ZV0508 suggested it is a worthy candidate for the treatment of 5T4 positive cancers. Further studies are under way to assess its pharmacodynamic and pharmacokinetic properties, which may give some insights into its advantages over the one with conventional mc‐MMAF.

## CONFLICT OF INTEREST

The authors have no conflict of interest.
