# Supplementary figures and images for: Antitumor activity of a 5T4 targeting antibody drug conjugate with a novel payload derived from MMAF via C‐Lock linker
Source: Cancer Med. 2019 Mar 7;8(4):1793–805. doi: 10.1002/cam4.2066 (PMC6488119; doi:10.1002/cam4.2066)

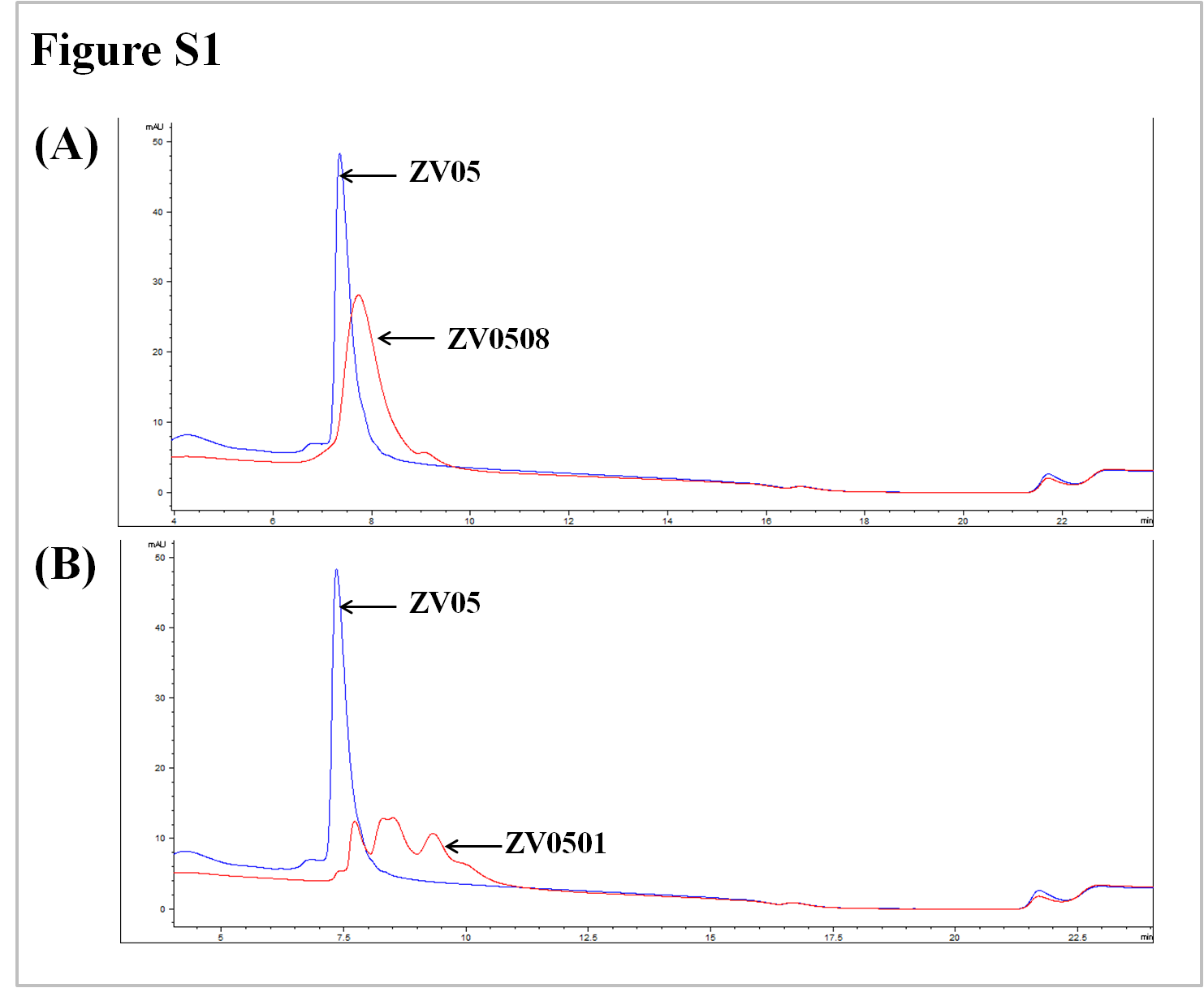

Supplement: Supplementary file 1 [file CAM4-8-1793-s001.tif]

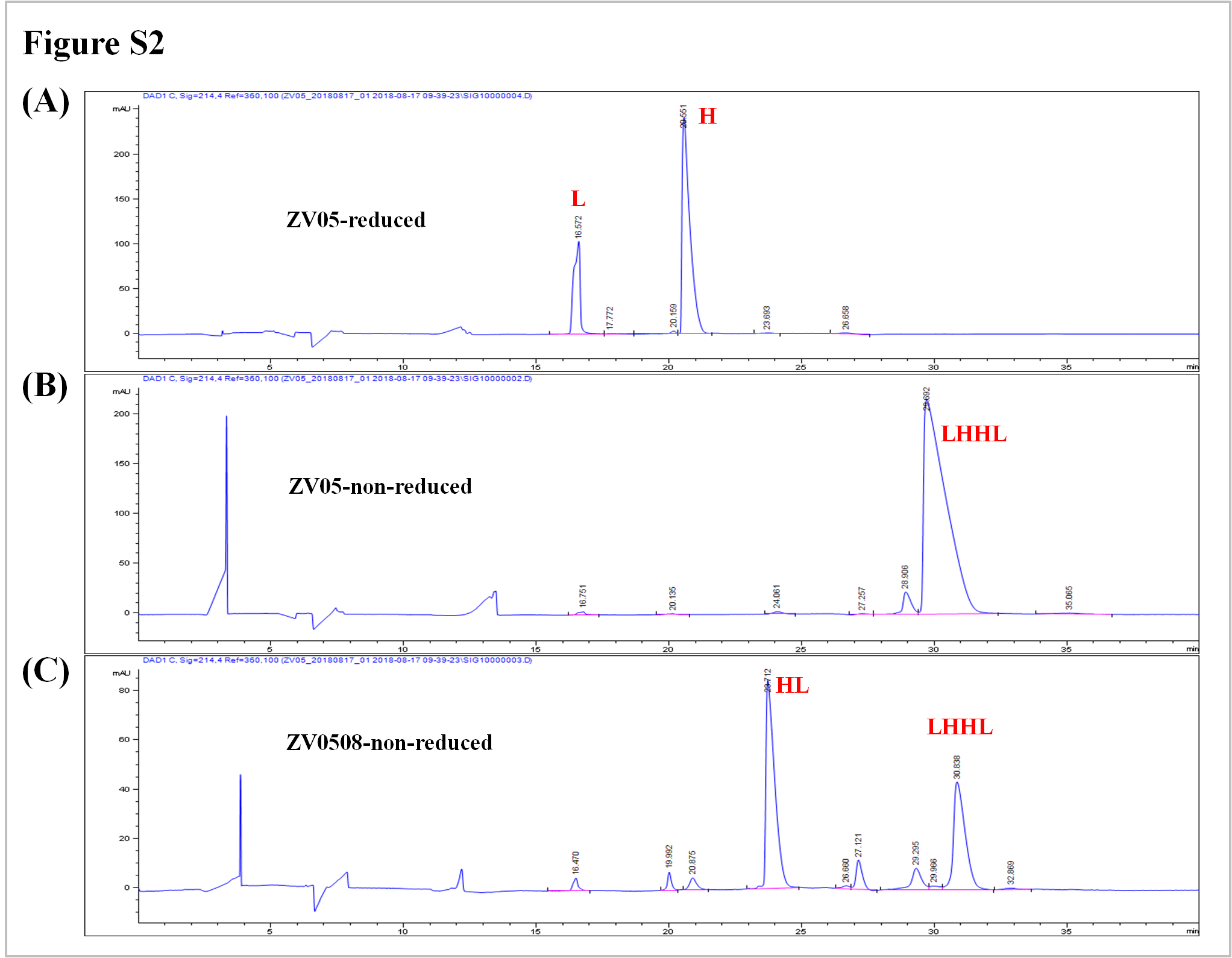

Supplement: Supplementary file 2 [file CAM4-8-1793-s002.tif]

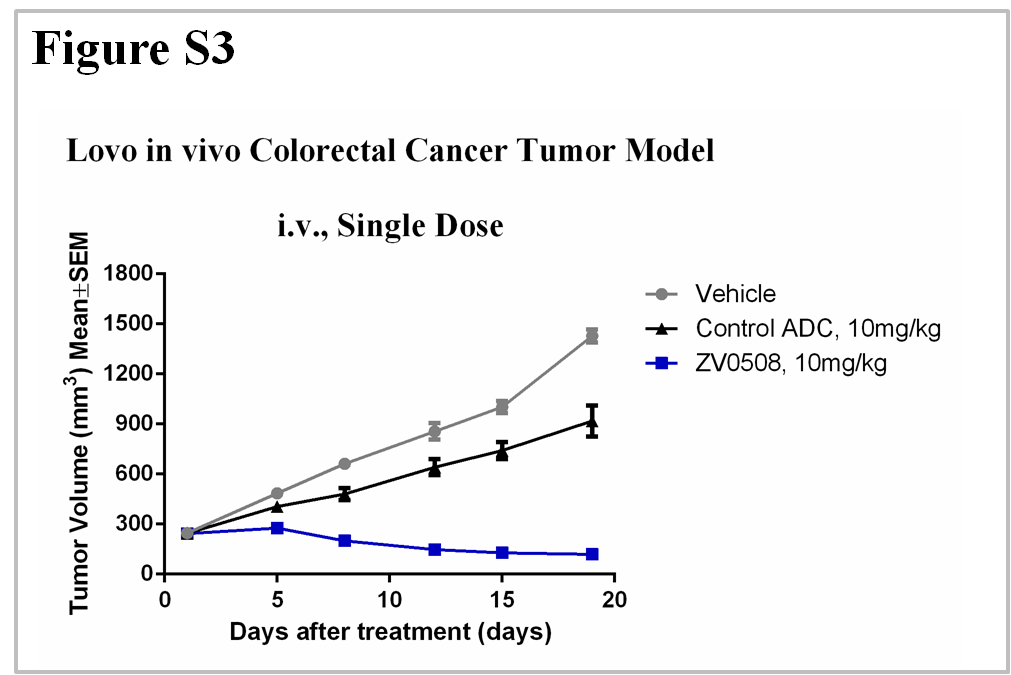

Supplement: Supplementary file 3 [file CAM4-8-1793-s003.tif]

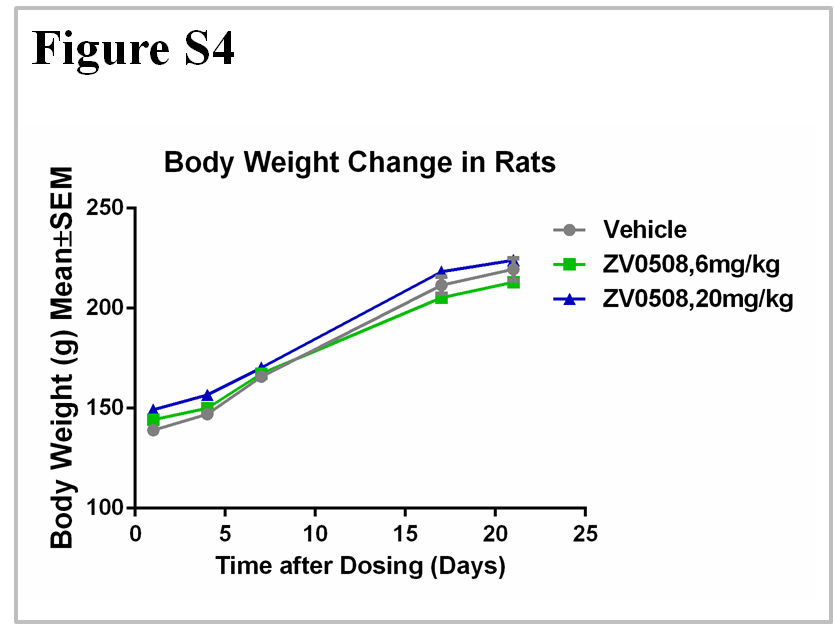

Supplement: Supplementary file 4 [file CAM4-8-1793-s004.tif]
